# The Southern Quarantine Conference

**Published:** 1905-12

**Authors:** 


### The Southern Quarantine Conference.

Following is the full text of the quarantine resolutions which were unanimously adopted by the conference of Southern States, recently held at Chattanooga:

QUARANTINE RESOLUTIONS.

WHEREAS, The experience of recent years, and especially the experience of this year, have demonstrated beyond cavil that the house mosquito, known as the Stegomyia fasciata, is the sole known cause of yellow fever epidemics and have demonstrated the futility and nuisance of many antiquated methods of quarantine hitherto resorted to, and the wisdom and necessity, in the interest of the public health and the public business, of uniform regulations to prevent the importation into the United States of yellow fever and its spread from State to State in the unfortunate event of its introduction; now, therefore, be it

*Resolved,* That we, delegates from Alabama, Arkansas, Florida, Georgia, Kentucky, Louisiana, Mississippi, Missouri, Maryland, North Carolina, South Carolina, Tennessee, Virginia and West Virginia, hereby respectfully request the Senate and House of Representatives in Congress assembled to enact a law whereby coast maritime and National frontier quarantine shall be placed exclusively under the control and jurisdiction of the United States government, and that matters of interstate quarantine shall be placed under the control and jurisdiction of the United States government, acting in co-operation with the several State Boards of Health.

We, furthermore, respectfully request that Congress shall make adequate appropriation to enforce and perfect the objects of this memorial and to stamp out as nearly as practicable the yellow fever-carrying mosquito in its breeding or living places in the United States, and by negotiating arrangements with the governments of Central and South America and the West India islands, in places where the said mosquito has its breeding places or exists in said countries.

*Resolved,* second, That we urge on the Legislatures of the several Southern States that they make quarantine regulations as nearly as possible in accord and conformity as hereinafter enacted.

We, furthermore, urge the Governors of the said several States with the above object in view specifically to call the attention of the Legislatures of their respective States to the wisdom and policy of this course.

